# Prognostic Analysis of Primary Breast Signet Ring Cell Carcinoma and Mucinous Breast Adenocarcinoma: A SEER Population-Based Study

**DOI:** 10.3389/fonc.2021.783631

**Published:** 2021-12-10

**Authors:** Song Wang, Yiyuan Zhang, Fangxu Yin, Xiangsheng Zhang, Zhenlin Yang, Xiaohong Wang

**Affiliations:** ^1^ Department of Thyroid and Breast Surgery, Binzhou Medical University Hospital, Binzhou, China; ^2^ Department of Reproductive Endocrinology, Affiliated Reproductive Hospital of Shandong University, Jinan, China

**Keywords:** signet ring cell, breast cancer, mucinous adenocarcinoma, SEER, prognosis

## Abstract

**Background:**

Primary breast signet ring cell carcinoma (SRCC) is a rare type of breast cancer with typical morphological characteristics, high aggressiveness, and poor prognosis. SRCC is different from mucinous breast adenocarcinoma (MBC). However, only a few studies have explored the clinicopathological features and prognosis of SRCC and MBC.

**Methods:**

Data retrieved from the Surveillance, Epidemiology, and End-Results (SEER) database (2004–2015) were used to explore the prognostic effect of clinicopathological features and treatment modalities on survival outcomes of SRCC and MBC patients. Kaplan–Meier plot analysis, multivariate Cox proportional risk model, propensity score matching (PSM), and subgroup analysis were performed.

**Results:**

A total of 167 patients with SRCC and 11,648 patients with MBC were included in the study. SRCC patients exhibited higher histological grade (*p* < 0.001), larger tumor volume (*p* < 0.001), higher rate of lymph node metastasis (*p* < 0.001), and higher frequency of distal metastasis (*p* < 0.001) compared with MBC patients. Cox proportional hazards regression analysis showed that SRCC patients had lower overall survival (OS) and breast cancer-specific survival (BCSS) compared with MBC patients. Subgroup survival analysis showed that the SRCC patients had lower OS and BCSS in subgroups including younger than 60 years old, white race, married, without chemotherapy, and received radiotherapy compared with the MBC patients in these subgroups. In addition, the SRCC patients had lower BCSS in subgroups including other races (including Asian or Pacific Islander and American Indian/Alaska Native), without surgery, and lymph node metastasis.

**Conclusion:**

The findings showed that primary breast SRCC patients have unique clinical characteristics and worse prognosis compared with MBC patients. Notably, different treatment methods resulted in different prognosis for SRCC and MBC types; therefore, SRCC patients should be distinguished from MBC patients to improve efficacy of treatment.

## Introduction

Breast cancer is the most common cancer type in the world and is associated with a high number of cancer-related deaths (1). Primary signet ring cell carcinoma (SRCC) is a rare variant of adenocarcinoma (2). SRCC was initially described as mucinous carcinoma by Saphir in 1941 (3). The classification of breast cancer groups by WHO in 2003 includes SRCC in mucinous carcinoma and other mucinous tumors (4). WHO breast tumor histological classification (Fourth Edition) changed the name of signet ring cell carcinoma to breast cancer with signet ring cell differentiation in 2012, indicating that signet ring cell differentiated breast cancer is no longer an independent category (5). Some studies report that SRCC mainly presents as high-grade lesions, and signet ring cells are associated with poor prognosis (6–8). Therefore, primary breast SRCC should be treated as an independent breast cancer type.

Mucinous breast cancer (MBC), also known as mucoid carcinoma or glial carcinoma, is a rare breast cancer subtype, accounting for 1.4%–5.2% of all breast types (9). MBC is characterized by slow and less metastasis (10). Previous studies report that MBC is a breast cancer type with good prognosis (11).

Studies initially classified SRCC as a special type of MBC (4). The two breast cancer types are characterized by mucus secretion. However, the prognosis of SRCC and MBC patients is different; thus, it is important to differentiate these subtypes. Although primary breast SRCC is highly malignant, only case reports and small sample retrospective studies have been conducted on SRCC owing to its low incidence (6–8, 10). Currently, there are no studies comparing primary breast SRCC and MBC subtypes. Therefore, the clinicopathological characteristics and prognosis of primary breast SRCC and MBC patients were compared in the current study using data retrieved from the SEER database (2004–2015). In addition, the effects of different treatment methods on the prognosis of SRCC and MBC patients were compared. Furthermore, the prognosis of SRCC and MBC patients under different subgroups was analyzed. The findings of the current study provide a basis for management and treatment of SRCC and MBC patients.

## Materials and Methods

### Data Resource and Study Population

All patients diagnosed with SRCC and MBC registered in the SEER database from 2004 to 2015 were included in the study. The location of primary cancer is limited to the breast (C50) according to the International Classification of Diseases for Oncology, 3rd Edition (ICD-O-3). Patients with SRCC (ICD-O-3 Code 8490/3) or MBC (ICD-O-3 Code 8480/3) were included in the current study. Patients were diagnosed through histological diagnosis, and breast cancer was their first or only cancer diagnosis. Patients lacking primary tumor and survival data were excluded. Demographic data and clinicopathological information patients in the two groups including age at diagnosis, race, marital status at diagnosis, tumor location, histological grade, tumor size, lymph node stage, metastasis status, estrogen receptor (ER) status, progesterone receptor (PR) status, surgical treatment of mastectomy or breast conserving surgery, radiotherapy, chemotherapy, cause of death, and survival (months) were retrieved. A total of 167 patients with SRCC and 11,648 patients with MBC met the inclusion criteria and were included in the study.

### Statistical Analysis

Patients were assigned to the MBC group and SRCC group. Baseline characteristics of participants were analyzed using Pearson’s chi square test or Fisher’s exact test to explore differences between the two groups. Uncorrected Kaplan–Meier curve and log rank test were used to determine the relationship between OS and BCSS with different histological subtypes and to explore the association between OS and BCSS of the two groups under different treatment methods. Propensity score matching (PSM) was used to eliminate bias and to further adjust the model for potential baseline confounding factors. Multivariate Cox proportional hazards model was used to determine the hazard ratio (HR) and 95% confidence interval (95% CI) of OS and BCSS in different subgroups stratified by histological type, to explore the potential risk factors of SRCC and MBC. Furthermore, the multivariate Cox proportional hazards model was used for subgroup analysis stratified by different clinical characteristics, and the HR and 95% CI of OS and BCSS under different clinical characteristics were compared. All tests were two-sided and *p* < 0.05 indicated statistical significance. R statistical software version 4.0.5 was used for all statistical analyses and generation of Kaplan Meier plots (http://www.R-project.org/). The forest plot is generated through the Sangerbox 3.0 (http://vip.sangerbox.com/).

## Results

### Baseline Characteristics of the Study Population

A total of 11,815 patients were registered in the SEER database from 2004 to 2015, namely, 11,648 MBC patients and 167 SRCC patients. The baseline characteristics of all patients are presented in **Table 1**. Analysis showed no significant difference in age and sex ratio between SRCC and MBC patients (*p* = 0.139; *p* = 0.121, respectively). Patients in the SRCC group exhibited larger tumor size (>T1, 40.1% vs. 31.2%, *p* < 0.001) and higher frequencies of lymph node metastasis (35.4% vs. 10.1%, *p* < 0.001) and distal metastasis (28.1% vs. 2.0%, *p* < 0.001) compared with patients in the MBC group. The findings showed higher number of patients with high-grade tumor in the SRCC group compared with the number of patients with high-grade tumor in the MBC group (>I, 69.4% vs. 34.4%, *p* < 0.001). Expression levels of ER and PR were lower in the SRCC group compared with the expression levels in the MBC group (74.9% vs. 93.2%, *p* < 0.001; 52.7% vs. 83.7%, *p* < 0.001, respectively). The operation rate in the SRCC group was lower than that in the MBC group (62.2% vs. 93.5%, *p* < 0.001). Similarly, the radiotherapy rate in the SRCC group was lower than that in the MBC group (29.9% vs. 46.6%, *p* < 0.001). Moreover, the rate of chemotherapy in the SRCC group was higher compared with the MBC group (37.7% vs. 14.1%, *p* < 0.001).

**Table 1:** Comparison of baseline characteristics of SRCC between MBC before PSM.

Characteristics	Patients, No. (%)		*p*-value
	MBC	SRCC	
**Age (years)**			0.139
<60	3,621 (31.1)	43 (25.7)	
≥60	8,027 (68.9)	124 (74.3)	
**Sex**			0.121
Female	11,600 (99.6)	165 (98.8)	
Male	48 (0.4)	2 (1.2)	
**Race**			0.004
Black	1,303 (11.2)	9 (5.4)	
White	8,946 (76.8)	149 (88.6)	
Others	1,313 (11.3)	9 (5.4)	
Unknown	86 (0.7)	1 (0.6)	
**Marital status**			0.945
Married	9,365 (80.4)	136 (81.4)	
Unmarried	1,698 (14.6)	23 (13.8)	
Unknown	585 (5.0)	8 (4.8)	
**Laterality**			<0.001
Left	5,997 (51.5)	78 (46.7)	
Right	5,622 (48.3)	73 (43.7)	
Other	29 (0.2)	16 (9.6)	
**Grade**			<0.001
I	6,105 (52.4)	8 (4.8)	
II	3,544 (30.4)	64 (38.3)	
III	443 (3.8)	49 (29.3)	
IV	22 (0.2)	3 (1.8)	
Unknown	1,534 (13.2)	43 (25.7)	
**T stage**			<0.001
T1	7,259 (62.3)	43 (25.7)	
T2	2,969 (25.5)	47 (28.1)	
T3	508 (4.4)	12 (7.2)	
T4	147 (1.3)	8 (4.8)	
Unknown	765 (6.6)	57 (34.1)	
**N stage**			<0.001
N0	9,884 (84.9)	81 (48.5)	
N1	911 (7.8)	28 (16.8)	
N2	155 (1.3)	12 (7.2)	
N3	117 (1.0)	19 (11.4)	
Unknown	581 (5.0)	27 (16.2)	
**M**			<0.001
M0	11,167 (95.9)	114 (68.3)	
M1	228 (2.0)	47 (28.1)	
Unknown	253 (2.2)	6 (3.6)	
**ER status**			<0.001
Positive	10,851 (93.2)	125 (74.9)	
Negative	199 (1.7)	28 (16.8)	
Unknown	598 (5.1)	14 (8.4)	
**PR status**			<0.001
Positive	9,748 (83.7)	88 (52.7)	
Negative	1,164 (10.0)	57 (34.1)	
Unknown	736 (6.3)	22 (13.2)	
**Surgery**			<0.001
No surgery	732 (6.3)	62 (37.1)	
Lumpectomy	7,218 (62.0)	53 (31.7)	
Mastectomy	3,671 (31.5)	51 (30.5)	
Unknown	270 (0.2)	1 (0.6)	
**Radiation**			<0.001
Yes	5,431 (46.6)	50 (29.9)	
No/Unknown	6,217 (53.4)	117 (70.1)	
**Chemotherapy**			<0.001
Yes	1,641 (14.1)	63 (37.7)	
No/Unknown	10,007 (85.9)	104 (62.3)	

SRCC, primary breast signet ring cell carcinoma; MBC, mucinous breast adenocarcinoma; PSM, reliability score matching.

### Survival Analyses of SRCC and MBC Groups

**Figure 1:**
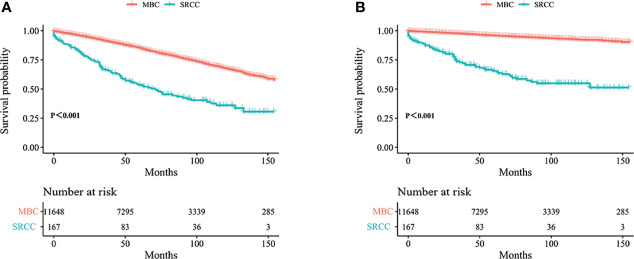
Kaplan–Meier curves: OS **(A)** and BCSS **(B)** among SRCC and MBC before PSM. OS, overall survival; BCSS, breast cancer-specific survival; SRCC, primary breast signet ring cell carcinoma; MBC, mucinous breast adenocarcinoma; PSM, Propensity Score Matching.

**Figure 2:**
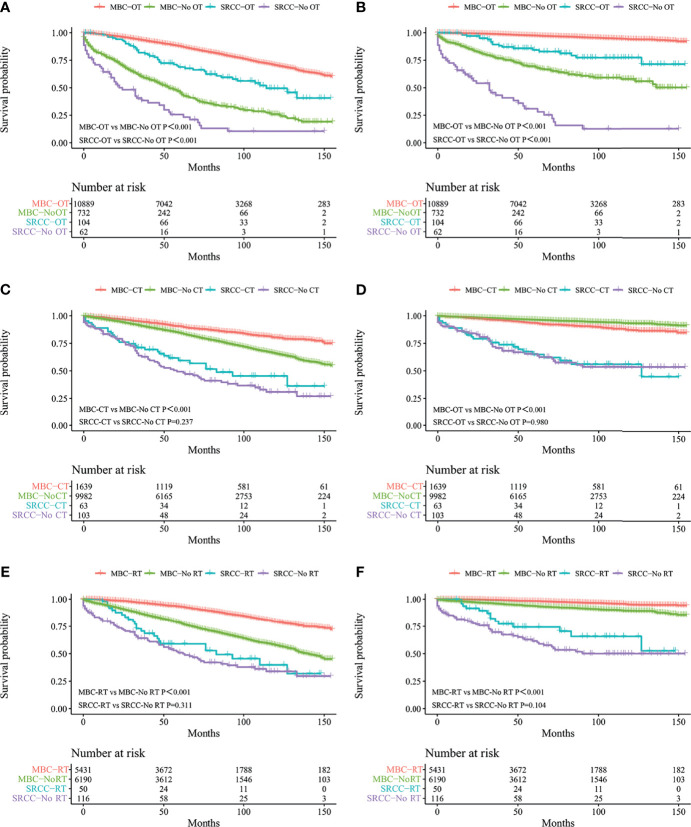
Kaplan–Meier curves: OS **(A)** and BCSS **(B)** in patients with MBC and SRCC receiving operation and without operation; OS **(C)** and BCSS **(D)** in patients with MBC and SRCC receiving chemotherapy and without chemotherapy; OS **(E)** and BCSS **(F)** in patients with MBC and SRCC receiving radiotherapy and without radiotherapy. OS, overall survival; BCSS, breast cancer-specific survival; SRCC, primary breast signet ring cell carcinoma; MBC, mucinous breast adenocarcinoma; OT, operation; No OT, without operation; CT, chemotherapy; No CT, without chemotherapy; RT, radiotherapy; No RT, without radiotherapy.

**Figure 3:**
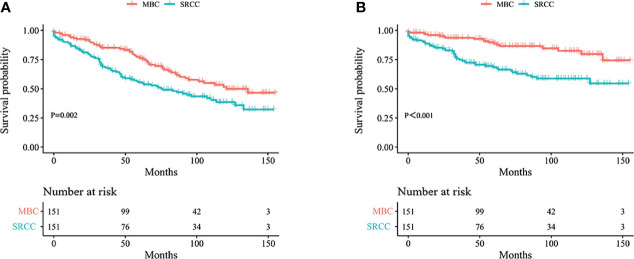
Kaplan–Meier curves: OS **(A)** and BCSS **(B)** among SRCC and MBC after PSM. OS, overall survival; BCSS, breast cancer-specific survival; SRCC, primary breast signet ring cell carcinoma; MBC, mucinous breast adenocarcinoma; PSM, Propensity Score Matching.

Kaplan–Meier analysis showed that OS and BCSS of patients in the SRCC group were significantly worse compared with the OS and BCSS of patients in the MBC group (**Figure 1**, both *p* < 0.001). The 5-year OS of the SRCC group and MBC group were 52.7% and 84.7%, respectively. The 5-year BCSS was 65.2% in the SRCC group and 95.9% in the MBC group. Surgery, chemotherapy, and radiotherapy are the conventional methods for systemic treatment of breast cancer; therefore, the OS and BCSS were compared in the following groups: operation group and without operation group; chemotherapy group and without chemotherapy group; radiotherapy group and without radiotherapy group. The results showed that surgery improved the OS of MBC patients and SRCC patients, respectively (both *p* < 0.001). In addition, the BCSS of the SRCC operation group was better than that of the without operation group (both *p* < 0.001), and the BCSS of the MBC operation group was also better than that of the without operation group. Interestingly, chemotherapy improved OS and BCSS in the MBC group (both *p* < 0.001). However, there was no significant difference in OS and BCSS between the SRCC group receiving chemotherapy and the SRCC group not receiving chemotherapy (*p* = 0.237 and *p* = 0.980, respectively). The analysis of the impact of radiotherapy on the prognosis of patients showed that the OS and BCSS of MBC patients receiving radiotherapy were better than those without radiotherapy (both *p* < 0.001). It should be noted that the OS and BCSS of SRCC patients receiving radiotherapy and SRCC patients not receiving radiotherapy were not statistically significant (*p* = 0.311 and *p* = 0.104, respectively). The findings on OS and BCSS are presented in **Figure 2**. The uneven baseline characteristics may have significantly affected survival results; therefore, a 1:1 PSM analysis was conducted to minimize baseline effects. A total of 151 MBC patients and 151 SRCC patients were matched. Analysis showed no significant difference in clinicopathological characteristics between the paired groups except for histological grade, estrogen receptor status, and progesterone receptor status characteristics between the paired groups (**Table 2**). Kaplan–Meier analysis showed that the clinical prognosis of SRCC patients was worse compared with the prognosis of MBC patients (**Figure 3**). Results for multivariable Cox proportional hazard regression models are presented in **Table 3**. After adjustments for age, race, T stage, N stage, and M stage, multivariable Cox proportional hazard regression analysis showed that OS was significantly worse in the SRCC group compared with the OS of the MBC group (HR = 1.320, 95% CI = 1.052–1.654, *p* = 0.016). In addition, the BCSS of SRCC patients was poor compared with the BCSS of the MBC group (HR = 1.931, 95% CI = 1.440–2.590, *p* < 0.001). After adjustments for age, race, lateral status, grade, T stage, N stage, M stage, surgery, radiation, and chemotherapy, multivariable Cox proportional hazard regression analysis also showed that patients in the SRCC group had significantly lower OS compared with the OS of the MBC patients (HR = 1.292, 95% CI = 1.028–1.625, *p* = 0.028). Patients in the SRCC group also showed a lower BCSS compared with MBC patients (HR = 1.671, 95% CI = 1.238–2.257, *p* = 0.001). The grade, ER, and PR levels were further adjusted after PSM. The findings showed a lower OS (HR = 1.842, 95% CI = 1.278–2.655, *p* = 0.001) and lower BCSS (HR = 3.271, 95% CI = 1.903–5.622, *p* < 0.001) for SRCC patients compared with the OS and BCSS for patients in the MBC group (**Table 3**).

**Table 2:** Comparison of baseline characteristics of SRCC between MBC after PSM.

Characteristics	Patients, No. (%)		*p*-value
	MBC	SRCC	
**Age (years)**			0.444
<60	46 (30.5)	40 (26.5)	
≥60	105 (69.5)	111 (73.5)	
**Sex**			1.000
Female	150 (99.3)	149 (98.7)	
Male	1 (0.7)	2 (1.3)	
**Race**			0.057
Black	16 (10.6)	9 (6.0)	
White	115 (76.2)	133 (88.1)	
Others	17 (11.3)	8 (5.3)	
Unknown	3 (2.0)	1 (0.7)	
**Marital status**			0.456
Married	112 (74.2)	121 (80.1)	
Unmarried	29 (19.2)	23 (15.2)	
Unknown	10 (6.6)	7 (4.6)	
**Laterality**			0.323
Left	64 (42.4)	76 (50.3)	
Right	78 (51.7)	65 (43.0)	
Other	9 (6.0)	10 (6.6)	
**Grade**			0.001
I	24 (15.9)	8 (5.3)	
II	66 (43.7)	64 (42.4)	
III	22 (14.6)	46 (30.5)	
IV	1 (0.7)	3 (2.0)	
Unknown	38 (25.2)	30 (19.9)	
**T stage**			0.425
T1	48 (31.8)	43 (28.5)	
T2	40 (26.5)	47 (31.1)	
T3	11 (7.3)	12 (7.9)	
T4	3 (2.0)	8 (5.3)	
Unknown	49 (32.5)	41 (27.2)	
**N stage**			0.192
N0	78 (51.7)	76 (50.3)	
N1	23 (15.2)	28 (18.5)	
N2	6 (4.0)	11 (7.3)	
N3	11 (7.3)	16 (10.6)	
Unknown	33 (21.9)	20 (13.2)	
**M**			0.063
M0	116 (76.8)	113 (74.8)	
M1	21 (13.9)	32 (21.2)	
Unknown	14 (9.3)	6 (4.0)	
**ER status**			0.001
Positive	128 (84.8)	115 (76.2)	
Negative	6 (4.0)	26 (17.2)	
Unknown	17 (11.3)	10 (6.6)	
**PR status**			0.013
Positive	101 (66.9)	86 (57.0)	
Negative	28 (18.5)	50 (33.1)	
Unknown	22 (14.6)	15 (9.9)	
**Surgery**			0.223
No surgery	39 (28.5)	47 (31.1)	
Lumpectomy	68 (45.0)	53 (35.1)	
Mastectomy	44 (29.1)	50 (33.1)	
Unknown	0 (0.0)	1 (0.7)	
**Radiation**			1.000
Yes	49 (32.5)	49 (32.5)	
No/Unknown	102 (67.5)	102 (67.5)	
**Chemotherapy**			0.626
Yes	53 (35.1)	49 (32.5)	
No/Unknown	98 (64.9)	102 (67.5)	

SRCC, primary breast signet ring cell carcinoma; MBC, mucinous breast adenocarcinoma. PSM, propensity score matching.

**Table 3:** Prognostic factors for overall survival (OS) and breast cancer-caused specific survival (BCSS) by multivariable Cox proportional hazard model.

Outcomes	SRCC HR (95% CI)	*p*-value
**Overall survival**		
Adjust I	1.320 (1.052, 1.654)	0.016
Adjust II	1.292 (1.028, 1.625)	0.028
PSM adjusted	1.842 (1.278, 2.655)	0.001
**Breast cancer-specific survival**		
Adjust I	1.931 (1.440, 2.590)	<0.001
Adjust II	1.671 (1.238, 2.257)	0.001
PSM adjusted I	3.271 (1.903, 5.622)	<0.001

SRCC, primary breast signet ring cell carcinoma; HR, hazard ratio; 95% CI, 95% confidence interval; PSM, propensity score matching. Adjusted I model adjusts for age, race, T stage, N stage, and M stage. Adjusted II model adjusts for age, race, laterality, grade, T stage, N stage, M stage, surgery, radiation, and chemotherapy. PSM-adjusted model adjusts for grade, ER, and PR.

### Subgroup Survival Analyses Between SRCC and MBC

**Figure 4:**
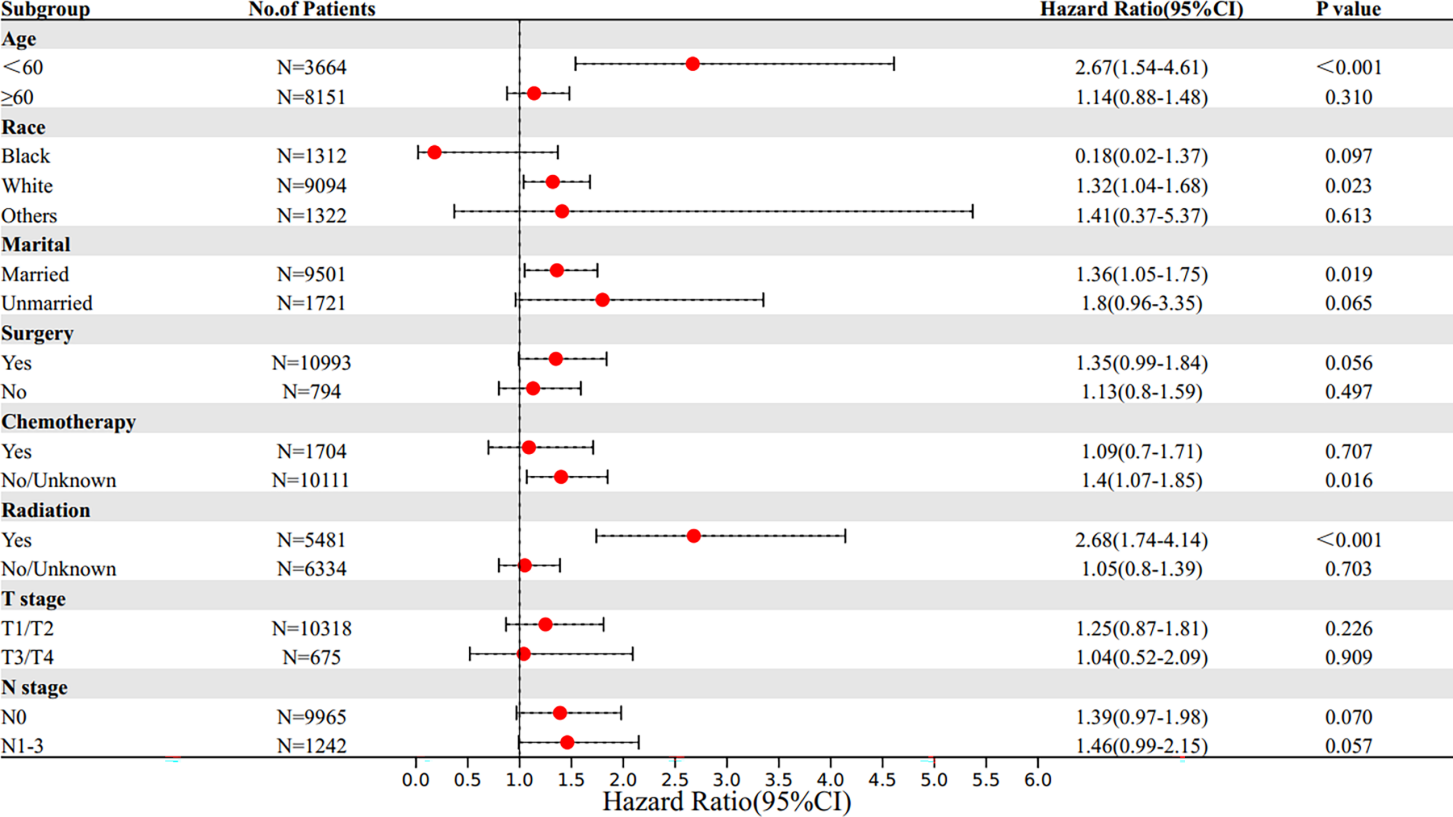
Subgroup multivariate Cox proportional hazards model comparing BCSS between SRCC and MBC. OS, overall survival; SRCC, primary breast signet ring cell carcinoma; MBC, mucinous breast adenocarcinoma.

**Figure 5:**
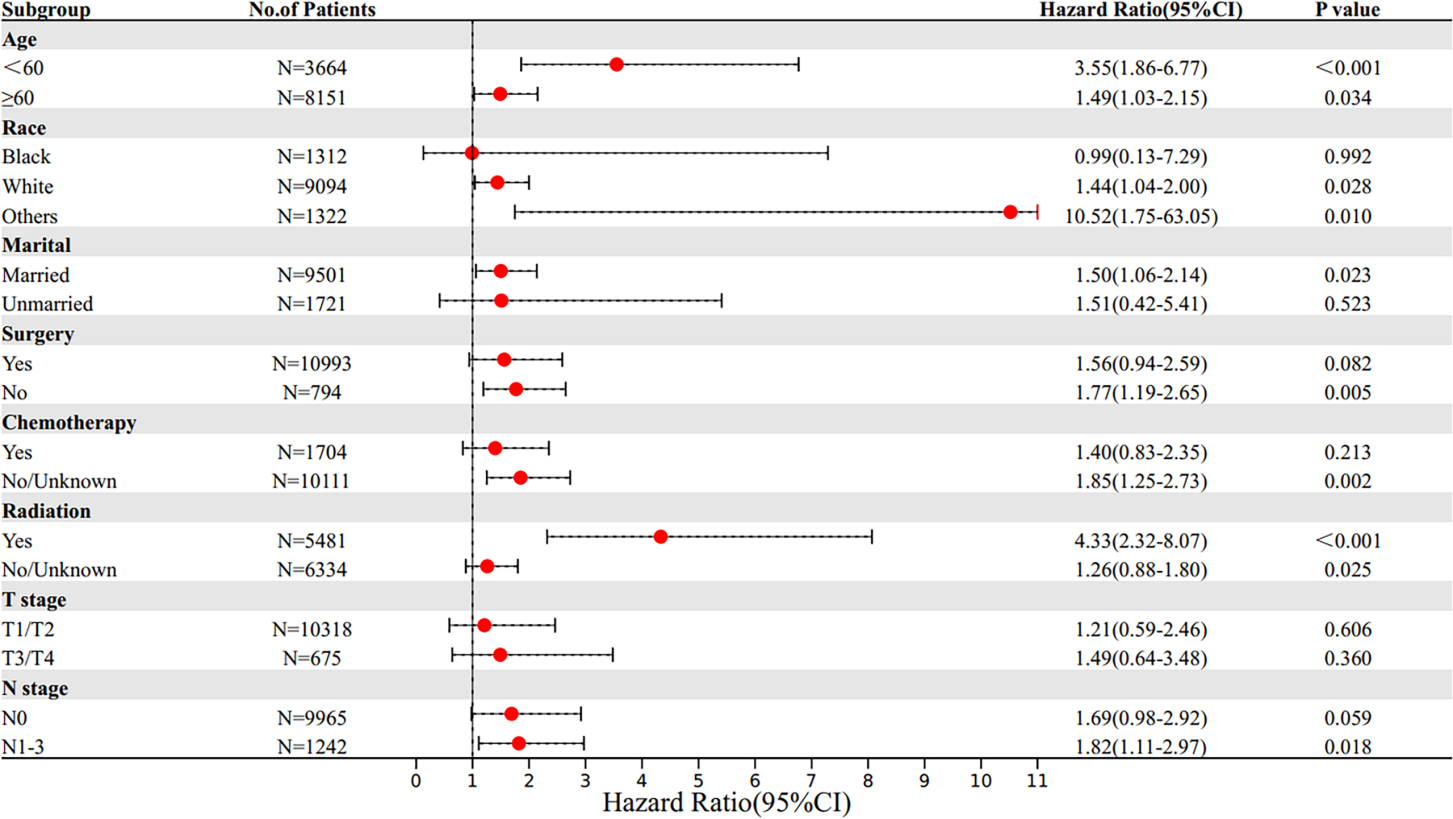
Subgroup multivariate Cox proportional hazards model comparing BCSS between SRCC and MBC. BCSS, breast cancer-specific survival; SRCC, primary breast signet ring cell carcinoma; MBC, mucinous breast adenocarcinoma.

Patients were stratified into subgroups according to the different clinical characteristics, and subgroup analysis was performed. Multivariate Cox proportional hazards model was used to determine the HR and 95% CI of OS and BCSS of different subgroups. Subgroup survival analysis results showed that the OS of SRCC patients was lower in those younger than 60 years old, white race, married, without chemotherapy, and received radiotherapy subgroups compared with the OS of MBC patients in these subgroups. In addition, the BCSS of SRCC patients was worse compared with that of MBC patients in those younger than 60 years old, white race, other races (including Asian or Pacific Islander and American Indian/Alaska Native), married, without surgery, without chemotherapy, received radiotherapy, and lymph node metastasis subgroups. Notably, the mortality risk of SRCC patients receiving radiotherapy was higher compared with that of MBC patients receiving radiotherapy, indicating that SRCC patients are less sensitive to radiotherapy compared with MBC patients (**Figures 4**, **5**).

## Discussion

The clinicopathological features and survival outcomes of primary breast SRCC patients and MBC patients were compared in this study. Primary SRCC is a highly malignant pathological type, most common in the stomach, followed by colon, esophagus, rectum, lung, pancreas, breast, bladder, small intestine, and gallbladder (12). Analysis of data from the SEER database shows that primary breast SRCC accounts for 1.5% of all SRCC cases, and the survival rate of patients with breast SRCC is higher compared with that of patients with gastric SRCC (13). The 2003 edition of WHO breast cancer classification indicates that SRCC is a specific type of mucinous carcinoma (4). The mucus distribution of signet ring cell carcinoma under a microscope is significantly different from that of mucinous carcinoma. Most of the mucus in SRCC is located in the cytoplasm, and high amounts of mucus in the cytoplasm pushes the nucleus to one side, thus forming a crescent shape. Mucus in breast mucinous carcinoma is located in the extracellular stroma, and cells float in the “mucus lake” forming sheet and nest shapes (14). The 2012 edition of WHO tumor classification reported the concept of cancer with signet ring cell differentiation. The cancer type is characterized by rich intracellular mucus, which pushes the nucleus to one side, resulting in characteristic signet ring cell morphology. Breast cancer with signet ring cell differentiation is no longer an independent breast cancer type (5). Clinical manifestation of this type of breast cancer is a painless and unclear boundary mass located in the outer and upper quadrant of the breast. It can occur in both the left and right sides of the breast. Notably, the small mass may not be detected by examination by hand or may present as a diffuse small nodule, which can be accompanied by changes in the nipple, such as nipple enlargement, ulceration, and fluid overflow. Changes in the skin can be manifested as local dimples and orange peel-like changes (15). This type of breast cancer is characterized by a late diagnosis; thus, most cases present with regional lymph nodes, and patients are prone to systemic metastasis to the stomach, uterus, lung, liver, and bone. Clinical manifestations of the patients include high malignancy, high invasion, and high metastasis, and the clinical prognosis is extremely poor (13). MBC is a rare type of breast cancer. It has a good prognosis, low lymph node metastasis rate, and low recurrence rate (11, 15). Although SRCC has similar pathological characteristics with MBC, and SRCC has been classified as mucinous carcinoma, the prognosis of the two cancer types is significantly different. It is necessary to distinguish SRCC from MBC. Most recent studies on SRCC are case reports as it is a rare type (2, 7). In addition, comparative studies between breast SRCC and MBC are few. The SEER database comprises a high amount of patient data and strong statistical efficiency, which makes the research based on SEER database have high clinical reference value.

We first compared the different clinical features of SRCC and MBC. The findings showed no significant difference in age and sex ratio between SRCC and MBC patients. In addition, the incidence of the two types of tumors was higher in elderly patients, which is consistent with findings from other studies (15, 16). Previous studies report that high-grade tumors were more common in the SRCC group compared with the MBC group (17, 18). Our findings indicated that the SRCC group was characterized by a later stage with advanced tumor stage, and higher incidence of lymph node metastasis and distal metastasis compared with the MBC group. Wu et al. (9) conducted a study with 11 patients with primary breast SRCC and 50 patients with MBC, and the finding showed that SRCC patients had more advanced disease and more frequent lymph node metastasis compared with MBC patients. Meanwhile, our results showed that ER and PR in the SRCC group were 74.9% and 52.7% positive, respectively. Chu et al. (19) performed a study comprising 21 cases of breast SRCC and reported that ER was 81% positive in signet ring cell carcinoma. Guo et al. (20) conducted a study with 14 cases of breast SRCC and reported that the positive rates of ER and PR were 71.4% (10/14) and 64.9% (9/14), respectively. These findings are consistent with our study. Expression levels of ER and PR were lower in the SRCC group compared with the expression levels in the MBC group. Moreover, surgery and radiotherapy rates of patients in the SRCC group were lower compared with the surgery and radiotherapy rates of patients in the MBC group. Consideration was given to the possibility that patients in the SRCC group had higher stage when they were diagnosed, and the surgery and radiotherapy were not effective treatment. In addition, the proportion of SRCC patients that underwent breast conserving procedure was significantly lower compared with the number of patients who underwent breast conserving in the MBC group. These findings indicate that breast conserving surgery should be carefully considered for SRCC patients owing to high aggressiveness of this cancer type. Furthermore, the chemotherapy rate of SRCC patients was higher compared with that of MBC patients. Reports on survival in SRCC patients have not been consistent. The Yale New Haven Medical Center pathology department (18) conducted a 4-year follow-up of 24 cases of breast cancer with signet ring cell differentiation from 1960 to 1979. The result showed that 9 patients (41.7%) survived out of 23 patients receiving treatment. Wu et al. (6) retrospectively analyzed 11 cases of breast signet ring cell carcinoma and 58 cases of mucinous adenocarcinoma of the breast. The 5-year OS of SRCC patients (54.5%) was significantly lower compared with that of MBC patients (88%). Recently, Wang et al. (21) conducted a study on 24 cases of simple signet ring cell carcinoma of the breast. The 5-year overall survival rate was 73.7%, and the 5-year specific survival rate of breast cancer was 78.3%. According to the Kaplan–Meier plot, the OS and BCSS of SRCC patients were significantly lower compared with the OS and BCSS of MBC patients. The 5-year OS and 5-year BCSS were 84.7% and 95.9%, respectively, in the MBC group, whereas the 5-year OS and 5-year BCSS were 52.7% and 65.2%, respectively, in the SRCC group. These findings indicate that the prognosis of SRCC patients is worse compared with that of MBC patients. Furthermore, the OS and BCSS performance of patients in the two groups was compared under different treatment methods. The results indicated that surgery significantly improves OS and BCSS of the two groups. In addition, chemotherapy improves OS and BCSS of MBC patients; however, SRCC patients treated with chemotherapy did not show an increase in OS and BCSS. Similar findings were observed for the group that had undergone radiotherapy. Radiotherapy improved the OS and BCSS of the MBC group, but did not improve the OS and BCSS in SRCC patients. Uneven baseline characteristics may result in significant effect on survival outcomes; thus, a 1:1 PSM analysis was performed to minimize baseline effects. A total of 151 MBC patients and 151 SRCC patients were matched after PSM (**Table 2**). There was no significant difference between the two groups after PSM except for histological grade, ER status, and PR status. Survival analysis showed that the clinical prognosis of SRCC patients was worse compared with that of MBC patients. In addition, multivariate Cox proportional hazard regression model analysis was used to compare the prognosis of SRCC and MBC patients. After adjustment of age, race, side, T stage, N stage, M stage, and treatment methods, multivariate Cox proportional hazards regression analysis showed that the OS rate for SRCC patients was lower compared with the OS of MBC patients. Analysis of BCSS showed that SRCC patients had poor prognosis compared with MBC patients. Additional adjustment analysis was performed on the mismatched baseline factors. Analysis after adjustment showed that SRCC patients had worse prognosis compared with MBC patients (**Table 3**).

In addition, subgroup survival analysis showed that the SRCC patients had lower OS and BCSS in subgroups including those younger than 60 years old, white race, married, and without chemotherapy compared with the MBC patients in these subgroups. In addition, the SRCC patients had lower BCSS in subgroups including other races (including Asian or Pacific Islander and American Indian/Alaska Native), without surgery, and lymph node metastasis. Interestingly, no matter OS or BCSS, the mortality risk of SRCC patients receiving radiotherapy was higher compared with that of MBC patients receiving radiotherapy, indicating that SRCC patients were less sensitive to radiotherapy compared with MBC patients. Previous studies suggested that SRCC is not sensitive to radiation (15, 18, 21). A previous study explored two cases of prostate signet ring cells mixed with urothelial carcinoma, and the finding showed that signet ring cells dominated after radiotherapy, implying that SRCC of the bladder had a poor response to radiotherapy (22). Ling et al. (23) found that the cancer-specific survival rate of SRCC in preoperative radiotherapy group was significantly lower than that of mucinous adenocarcinoma. The prognosis of patients with SRCC was significantly poorer in the preoperative radiotherapy setting of locally advanced rectal cancer. Furthermore, the mortality risk of SRCC patients receiving chemotherapy was higher than that in MBC patients receiving chemotherapy. However, the prognosis of SRCC patients and its sensitivity to specific chemotherapy regimens are still controversial. Most studies have not determined that SRCC is sensitive to chemotherapy (24, 25), and its poor prognosis may be attributed to diagnosis at advanced stage (12). However, recent studies have shown that SRCC is sensitive to chemotherapy. Hugen et al. (26) reported that chemotherapy can improve the survival rate of patients with colorectal signet-ring cell carcinoma, which is consistent with our conclusion. Subgroup survival analysis showed no significant difference in the risk of death in SRCC patients who underwent surgery compared with MBC patients who underwent surgery, while survival curves and previous studies (6, 27) suggest that surgery significantly improves survival in both types of patients. Our study found that surgery provides a higher survival benefit for SRCC patients than for MBC patients. So far, our study explored the clinical characteristics and prognosis of primary breast signet ring cell carcinoma and mucinous breast adenocarcinoma using a large sample size and the long follow-up time. We suggest that an advanced combination therapy should be considered once breast cancer is found to contain SRCC components.

However, the study had some limitations. SEER database is a large database containing 18 cancer registries in the United States, and data entry errors and bias are inevitable in the database itself. Patient follow-up information did not include disease-free survival date. Furthermore, the database does not provide data on endocrine and targeted therapy and specific chemotherapy regimens for patients; thus, further studies should explore the effects of these treatments on SRCC and MBC types. With the expansion of SEER database, further studies can provide more comprehensive and accurate information for the prognostic factors of primary breast SRCC.

## Conclusion

Primary breast SRCC patients have unique clinical characteristics and worse prognosis compared with MBC patients. Differences in survival rates were observed even after adjusting for basic demographic and clinicopathological features as well as treatment modalities. Notably, different treatment methods resulted in different prognosis for SRCC and MBC types; therefore, SRCC patients should be distinguished from MBC patients to improve efficacy of treatment.

## Data Availability Statement

Publicly available datasets were analyzed in this study. These data can be found here: https://seer.cancer.gov/data/.

## Ethics Statement

Ethical review and approval was not required for the study on human participants in accordance with the local legislation and institutional requirements. Written informed consent for participation was not required for this study in accordance with the national legislation and the institutional requirements.

## Author Contributions

SW is responsible for the design of the project, and YZ is responsible for data analysis. All authors participated in the writing of the final manuscript and approved the final submission.

## Funding

This study is supported by grants from the China National Natural Science Youth Fund Committee (Grant No. 81902702), the Shandong Province Clinical Key Specialist Project Construction Fund (20110731250), and the establishment and demonstration of regional collaborative graded diagnosis and treatment service model and clinical path of domestic innovative digital diagnosis and treatment equipment (2018YFC0114705).

## Conflict of Interest

The authors declare that the research was conducted in the absence of any commercial or financial relationships that could be construed as a potential conflict of interest.

## Publisher’s Note

All claims expressed in this article are solely those of the authors and do not necessarily represent those of their affiliated organizations, or those of the publisher, the editors and the reviewers. Any product that may be evaluated in this article, or claim that may be made by its manufacturer, is not guaranteed or endorsed by the publisher.
